# The Emerging *TNNT3* Spectrum: From Distal Arthrogryposis to Congenital Myopathy

**DOI:** 10.1155/humu/1785045

**Published:** 2025-12-28

**Authors:** Nami Altin, Kamel Mamchaoui, Jessica Ohana, Anne Bigot, Beatrice Corradi, Luca Maragliano, Francesca Madia, Marzia Ognibene, Mohammad Sadegh Shams Nosrati, Dario Paladini, Michele Iacomino, Asma Rashid, Olaf Bodamer, Susana Quijano-Roy, Jaya Punetha, Valeria Capra, Federico Zara, Capucine Trollet, Marcello Scala

**Affiliations:** ^1^ Sorbonne Université, INSERM, Institute of Myology, Centre of Research in Myology, Paris, France, sorbonne-universites.fr; ^2^ Center for Synaptic Neuroscience and Technology, Istituto Italiano di Tecnologia, Genoa, Italy, iit.it; ^3^ Department of Experimental Medicine, University of Genova, Genoa, Italy, unige.it; ^4^ Department of Life and Environmental Sciences, Polytechnic University of Marche, Ancona, Italy, univpm.it; ^5^ Medical Genetics Unit, IRCCS Istituto Giannina Gaslini, Genoa, Italy, gaslini.org; ^6^ Department of Neurosciences, Rehabilitation, Ophthalmology, Genetics, Maternal and Child Health, Università Degli Studi di Genova, Genoa, Italy, unige.it; ^7^ Fetal Medicine and Surgery Unit, Department Mother and Child, IRCCS Istituto Giannina Gaslini, Genoa, Italy, gaslini.org; ^8^ Division of Genetics and Genomics, Department of Medicine, Boston Children’s Hospital/Harvard Medical School, Boston, Massachusetts, USA, harvard.edu; ^9^ Garches Neuromuscular Reference Center, Child Neurology and ICU Department, APHP Raymond Poincare University Hospital (UVSQ Paris Saclay), Garches, France; ^10^ GeneDx, Gaithersburg, Maryland, USA, genedx.com; ^11^ Clinical Genomics and Genetics Unit, IRCCS Istituto Giannina Gaslini, Genoa, Italy, gaslini.org

**Keywords:** congenital myopathy, distal arthrogryposis, loss of function, recessive variants, *TNNT3*, troponin T3

## Abstract

Distal arthrogryposis (DA) is a group of nonprogressive congenital muscular disorders affecting distal limb joints, without concurrent neuromuscular disease. Ten different types of DAs are known, with many different genes involved. Dominant variants in *TNNT3* (MIM ∗600692) cause DA type 2B2 (MIM #618435), a severe condition featuring dysmorphism, distal contractures, and deformities of hands and feet. *TNNT3* encodes the fast skeletal troponin T, an essential component of the troponin complex that is necessary for calcium‐coupled contraction initiation in the striated muscle. Recently, homozygous splicing variants in *TNNT3* have been reported in two subjects with a distinctive congenital myopathy, only partially overlapping DA2B2. However, no functional evidence was provided. In this study, we investigated two patients presenting with myopathic conditions at different ends of the *TNNT3* spectrum. One subject showed DA, whereas the second displayed a severe congenital myopathy featuring hypotonia, DA, and dysmorphism. Through exome sequencing, we identified the de novo missense change p.(Arg63His) in Subject #1 and biallelic *TNNT3* variants in Subject #2, featuring a splicing and a stop gain variant. The p.(Arg63His) was predicted to affect the stability of troponin T3 in silico, and we confirmed this by western blot. Then, employing different biochemical approaches, we showed that the truncated variants identified in #2 (p.[Tyr13∗] and c.480+5G>A) lead to loss of the full‐length protein. Our findings refine and expand the *TNNT3* genotype–phenotype spectrum, suggesting that recessive *TNNT3*‐related congenital myopathy should be considered a discrete entity caused by biallelic loss‐of‐function variants.

## 1. Introduction

Distal arthrogryposis (DA) encompasses a group of autosomal dominant muscular disorders characterized by nonprogressive congenital contractures affecting the distal limb joints in the absence of a concomitant neuromuscular disease [1, 2]. Although individually rare, these conditions collectively affect up to one in 5000 new births, with a significant impact on patients’ lives [1–3]. The diagnosis of DA is based on the presence of more than two of the following features: absent flexion finger creases, camptodactyly, thumb adduction, ulnar deviation, overlapping or flexion fingers and toes, and valgus, vertical talus, and/or talipes equinovarus [1]. In severe cases, proximal joints may also be affected, leading to complications such as congenital hip dislocation, stiff elbows, and scoliosis [1–3].

Ten different types of DA have been defined (DA1–10) based on the proportion of shared features [1]. Although these disorders are quite heterogeneous, distinctive DAs show overlapping clinical features and underlying pathophysiological mechanisms [3]. Variations in many muscular genes have been involved in the pathogenesis of DAs, including myosin Heavy Chain 3 (*MYH3*, MIM ∗160720), troponin I Type 1 (*TNNI1*, MIM ∗191042), troponin I Type 2 (*TNNI2*, MIM ∗191043), and fast skeletal muscle TNNT (*TNNT3*, MIM ∗600692) [2, 4–9]. Among these, de novo or dominant variants in the *TNNT3* gene cause a severe form of DA known as DA2B2 (MIM #618435), a well‐known condition characterized by facial dysmorphism associated with contractures and deformities affecting the hands and feet [5, 10, 11]. Conversely, the report of biallelic variants in *TNNT3* in association with myopathic features still remains limited to a few cases [12–14].

Multiple isoforms of troponin T exist as a result of alternative splicing processes involving different members of a multigene family [15]. The *TNNT3* gene encodes troponin T fast (TnT fast), which is the ~30 kDa fast skeletal muscle isoform of TnT [15, 16]. This is part of the troponin complex, together with TnC and TnI isoforms, which plays a crucial role in the contraction of striated muscle [15, 16]. This process is initiated by the binding of intracellular calcium to the troponin complex located in the thin filaments, where the TnT subunit binds the complex to tropomyosin [15, 16]. As such, TnT contributes to regulating calcium‐triggered contraction and relaxation of muscle fibers [15, 16]. In mice, troponins are expressed in smooth muscle and play a pivotal role in guaranteeing normal growth and breathing for postnatal survival [17]. In humans, deleterious variants in genes encoding skeletal troponins can compromise sarcomere function, leading to a severe distortion of skeletal muscle mechanics and resulting in a spectrum of congenital myopathies [18]. In particular, the dysfunction of TnT3 resulting from deleterious de novo or dominant genetic variants has been associated with a dominant form of DA with peculiar facies and multiple deformities of the hands and feet [1–3].

Different genes involved in muscular disorders have been linked to variable myopathy phenotypes through either dominant or recessive patterns [19]. Indeed, deleterious *TNNT3* variants may cause dominant DA2B2 but also an emerging recessive congenital myopathy [2, 5, 12, 13, 20]. DA2B is characterized by a triangular face with a small mouth, hip dislocation, hand deformities (camptodactyly, clinodactyly, overlapping and tapered fingers, abnormal digital flexion creases, ulnar deviation of fingers and wrists, and adducted thumbs), and foot abnormalities (vertical talus, varus or equinovalgus deformity, metatarsus varus, clubfoot, camptodactyly, and short toes) [2, 5, 20]. This disorder shows some overlap with DA1, another arthrogryposis caused by variants in *TPM2* (MIM ∗190990), *TNNI2*, or even *TNNT3*. Thus, rather than distinct conditions, DA1 and DA2B are extremes of the same phenotypically and genetically heterogeneous condition [2, 5, 20]. Recessive *TNNT3* variants have emerged to cause a congenital myopathy characterized by progressive severe weakness, hypotonia, hypotonic facies, contractures, laxity, scoliosis, and need for respiratory support [12, 13]. Muscle biopsy may show nemaline rods or variable defects in fiber size and differentiation associated with fatty infiltration [12, 13]. This recessive myopathy should be considered a distinct *TNNT3*‐related clinical entity not only for the different genotypes of affected individuals but also because of the progressive course and the presence of clinical manifestations that are absent in DA2B2, such as severe weakness, hypotonia, and abnormal muscle histology [2, 5, 12, 13, 20].

In this study, we identified two novel subjects presenting with myopathic conditions placed at the different ends of the *TNNT3* spectrum, featuring dominant DA and recessive congenital myopathy. Through different biochemical approaches, we assessed that recessive *TNNT3* variants severely impact protein expression, supporting a distinctive loss of function mechanism in the recessive *TNNT3*‐related congenital myopathy.

## 2. Methods

### 2.1. Patient Enrolment and Clinical Assessment

This study adheres to the principles set out in the Declaration of Helsinki. The study was approved by the Research Ethics Committees of the Gaslini Children’s Hospital (Comitato Etico della Regione Liguria [163/2018]). Informed consent was obtained by the enrolled subjects or their parents or guardians. Patients were thoroughly evaluated by pediatricians and medical geneticists with expertise in neuromuscular disorders.

### 2.2. Exome Sequencing (ES)

ES was performed for both patients as previously described [21]. Variants were filtered out for minor allele frequency (MAF) ≤ 0.01 in genomic databases (gnomAD, V4.1.0, https://gnomad.broadinstitute.org). Then, in silico tools were employed to predict the impact of candidate variants on protein structure and function, including CADD score (https://cadd.gs.washington.edu), MutationTaster (http://www.mutationtaster.org), Mutation Assessor (http://mutationassessor.org/r3/), PolyPhen‐2 (http://genetics.bwh.harvard.edu/pph2/), and SpliceAI (https://spliceailookup.broadinstitute.org) [22]. All variants were classified according to the American College of Medical Genetics and Genomics and the Association for Molecular Pathology (ACMG/AMP) guidelines. Sample relatedness analysis was also performed. Further details are available in supporting information. *TNNT3* variants are reported according to the canonical NM_006757.4 RefSeq transcript, corresponding to Isoform 1 (NP_006748.1) (https://www.ncbi.nlm.nih.gov/nuccore/NM_006757.4).

### 2.3. 3D Modeling of *TNNT3* Variants

The sequence for the human wild‐type (wt) TNNT3 protein was retrieved from the NCBI databank (record NP_006748.1) (https://www.ncbi.nlm.nih.gov/protein/), and the structural predictions were performed with AlphaFold2 (AF2) Colab [23] and the D‐I‐TASSER [24] algorithm. The impact of single‐point missense variants on structure stability was quantified by calculating the difference in the Gibbs free energy of unfolding (*Δ* *Δ* *G*) between the mutated structure and its wt, using a method relying on an antisymmetric neural network, ACDC‐NN [25]. Negative *Δ* *Δ* *G* values indicate destabilization of the structure.

### 2.4. Plasmid Constructions

To generate a TNNT3 construction fused to GFP, the human TNNT3 cDNA (NM_001042781) was amplified (primers available in supporting information) and introduced into the pEGFP‐N1 vector (Clontech) between *Hind*III and *Age*I restriction sites. TNNT3‐207 (NM_001042781.3) is the most abundant transcript expressed in muscle cells but is highly homologous to the canonical NM_006757.4 transcript used for diagnostics, including the mutations highlighted in this study (Figure S1).

To generate a TNNT3 minigene plasmid fused to GFP, the human TNNT3 genomic region between Exon 10 and Exon 14 was introduced into the TNNT3 cDNA sequence and cloned into the pEGFP‐N1 plasmid. To do so, the NEB builder HiFi DNA assembly cloning kit (New England Biolabs) was used. Three TNNT3 fragments were amplified (primers available in supporting information): TNNT3 cDNA from the start codon to Exon 10, TNNT3 gDNA from Intron 10–11 to Intron 13–14, and finally, TNNT3 cDNA from Exon 14 to the last codon before the stop codon. The fragments were assembled into pEGFP‐N1 linearized by *Age*I and *EcoR*I, following the recommended ratio of the manufacturer.

Site‐directed mutagenesis was performed using the Q5 site‐mutagenesis kit (New England Biolabs) to introduce the c.39C>G, c.187C>T, and c.188G>A variants in pCMV‐TNNT3‐eGFP and the c.480+5G>A variant into pCMV‐TNNT3 minigene–eGFP (primers available in supporting information). Sequencing was done using the Mix2Seq Kit from Eurofins (Sanger sequencing).

### 2.5. Cell Culture and Transfection

Human immortalized myoblasts AB1190 were grown in KMEM: 199 Medium (Life Technologies) and DMEM (Life Technologies) in a 1:4 ratio supplemented with 20% fetal calf serum (Life Technologies), 5 ng/mL human epithelial growth factor (Life Technologies), 0.5 ng/mL bFGF, 0.2 *μ*M dexamethasone (Sigma‐Aldrich), 50 *μ*g/mL fetuin (Life Technologies), and 5 *μ*g/mL insulin (Life Technologies) in a 5% CO_2_ incubator at 37°C. HEK293T cells were grown in DMEM (Life Technologies) supplemented with 10% fetal calf serum (Life Technologies). Transfection of human immortalized myoblasts was performed with Amaxa II nucleofector (Lonza) using Basic Nucleofector Kit for Primary Mammalian Smooth Muscle Cells (Lonza) following the manufacturer’s instructions: 1 million cells were mixed with 2 *μ*g of plasmid and transfected using A‐033 program. Transfection of HEK293T cells was performed with Lipofectamine 2000 (Thermo Fisher) according to the manufacturer’s instructions. Cells were harvested 48 h after nucleofection for RNA or protein extraction.

### 2.6. Western Blot

Cell lysates were prepared by homogenizing tissue in RIPA solution (NaCl 0.15 M, HEPES 0.05 M, NP‐40 1%, sodium deoxycholate 0.5%, SDS 0.10%, and EDTA 0.05 M) with protease inhibitor cocktail (cOmplete, Roche Diagnostics). Proteins were separated on 4%–12% Bis‐Tris gel (Invitrogen) and transferred onto a nitrocellulose membrane (Hybond ECL membrane; Amersham Biosciences), which was blocked by incubation in 5% BSA diluted in 1X Tris‐buffered saline 0.1% Tween 20 (TBST). The membrane was probed with primary antibodies raised against GFP (Takara; ref 632592; 1:1000) as a loading control in 2.5% BSA in TBST. The membrane was further incubated with HRP‐conjugated antibodies (Jackson ImmunoResearch; 1:40,000). Immunoreactive bands were detected with enhanced chemiluminescence reagent (ECL; Amersham Biosciences) and signals visualized by exposing the membrane.

### 2.7. Visualization of TNNT3‐GFP Expression and Image Acquisition

Cells were cultivated on a 14‐mm cover slip coated with 0.5% gelatin (Sigma). Cells were washed once with PBS and fixed for 10 min with paraformaldehyde 4% PBS. Cells were washed with PBS three times for 5 min and incubated for 10 min with Hoechst (Thermo Fisher; 1/5000 in PBS). Cells were washed again three times for 5 min with PBS, and cover slips were mounted on a glass slide with Dako Fluorescence Mounting Medium (Agilent). Images were taken and visualized using an Apotome 2 microscope (ZEISS), digitalized using a Hamamatsu digital camera C11440 ORCA‐Flash4.0 LT, and analyzed using the Zen 3.0 image analysis system (ZEISS) and ImageJ 1.53t (http://imagej.nih.gov/ij).

### 2.8. RT‐PCR

Cell lysate was prepared by adding TRI reagent (Sigma) to cells. RNA was then extracted following the manufacturer’s instructions. RNA (1 *μ*g for cell pellet) was reverse transcribed using M‐MLV reverse transcriptase (Invitrogen) according to the manufacturer’s instructions. The splicing of *TNNT3* mRNA was observed with 1 *μ*L of cDNA for PCR using Reddy mix polymerase (Thermo Scientific) according to the manufacturer’s instructions and primers located within *TNNT3* Exon 15, *TNNT3* Exon 16, and *TNNT3* Exon 17 (primers available in supporting information). The reaction mixture was heated to 98°C for 1 min and followed by 30 PCR cycles: 15 s at 94°C, 30 s at 60°C, and 40 s at 72°C followed by a last elongation step at 72°C for 5 min. PCR products were then migrated in 2% agarose gel. Pictures of the gels were then analyzed with ImageJ in order to extract the intensities of each band.

## 3. Results

### 3.1. Clinical Data

The study involved two patients from unrelated families, presenting with significant myopathic manifestations (Figure 1a).

Figure 1Summary of genetic and clinical findings and analysis of Arg63 variants in human TNNT3 protein. (a) Pedigrees of the reported families (Family I and Family II) and clinical features of Subjects #1 and #2. The pedigrees show that biallelic *TNNT3* variants segregate with the clinical phenotype in affected individuals, while unaffected parents are healthy carriers. In Subject #1 (left), ultrasonography shows severe distal contractures affecting the feet and the hands. In the left hand, there are contractions in flexion of the third and fourth digits, as well as contractures in extension of the second and fifth digits. Flexion contractures affected all the digits of the right hand. There is severe bilateral clubfoot with significant plantar contracture. In Subject #2 (right), at the age of 4 years, there are hypotonic facies with micrognathia and posteriorly rotated ears with thin earlobes. Additional features include bilateral vertical talus associated with camptodactyly, brachydactyly, and short toes with broad halluces. Hand deformities consist of bilateral clinodactyly, camptodactyly, and brachydactyly. Additionally, there are short middle fingers, ulnar deviation of fingers and wrists, adducted thumbs, and abnormal digital flexion creases. (b) Schematic drawing of the TNNT3 gene (NM_006757.4) and protein (NP_001036245.1), showing the localization of previously reported variants (below) and the variants identified in our patients (above). Splicing variants affect Introns 12 and 14, whereas missense changes affect Exons 4 and 10. Interestingly, none of the missense variants is localized within the troponin functional domain. The truncating p.(Tyr13∗) variant is localized very proximally in the protein. (c) Structural prediction of the TNNT3 human protein. AF2‐TNNT3 is depicted as (A) cartoon and colored by pLDDT confidence score: Blue areas indicate a high score, while orange ones a low score. Alpha carbons of M1, R63, and K258 are shown as spheres. (B) Side chains of positively charged residues Q60‐K66. (C) Single point substitutions at Position 63. (d) Analysis of Arg63 variants in vitro. After transfection in HEK293T cells of wild type (wt) or R63H and R63C variants of TNNT3 fused to GFP expression plasmids, GFP detection revealed several isoforms including two main ones at 57.7 and 49.9 kDa for all transfections. Quantification of the two main bands of TNNT3‐GFP showed an increased stability with the R63H and R63C variants. *T*‐test  ^∗^ *p* < 0.05;  ^∗∗^ *p* < 0.01.

**Figure:**
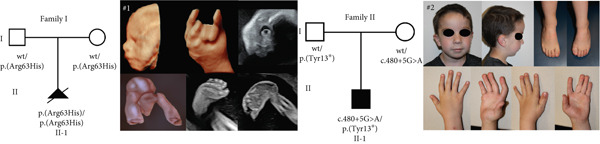
(a)

**Figure:**
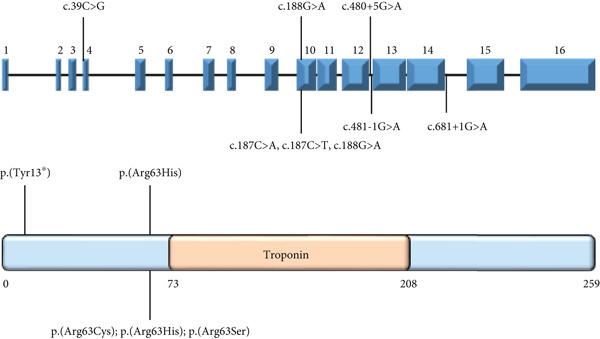
(b)

**Figure:**
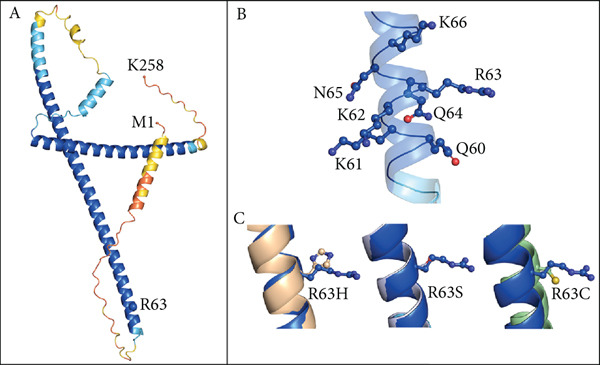
(c)

**Figure:**
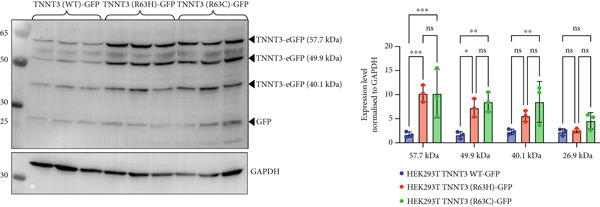
(d)

Subject #1 is an aborted fetus conceived by nonconsanguineous healthy parents. Pregnancy was uneventful until the ultrasound fetal examination performed at 20 + 4 gestation weeks. While no other gross morphological defects were observed, the evaluation of the musculoskeletal district revealed multiple malformations, suggestive of a DA. The fetus showed bilateral clubfoot with significant plantar contracture. There were significant contractures in both hands, either in flexion or extension (Figure 1a). No additional malformations were observed. After the identification of these abnormalities, the couple was informed of the likely diagnosis of DA, and they opted for termination of pregnancy.

Subject #2 is a 2‐year‐old proband born to a couple of nonconsanguineous parents. Family history was significant for a history of bilateral clubfeet in the father (Figure S2). Similar abnormalities were absent in other family members. Pregnancy was complicated by the identification of a bicornate uterus and severe polyhydramnios. Additionally, on fetal ultrasonography, multiple anomalies were observed, featuring microretrognathia, bilateral clubfeet, and clenched hands. The patient was delivered at 37 + 2 gestation weeks through cesarean section. Perinatal course was complicated by respiratory distress requiring ventilatory support, with occasional apneic and bradycardic spells, and feeding difficulties needing nasogastric tube feeds. Growth parameters at birth were normal except for length (44 cm, −3.11 SDS). Physical exam showed axial hypotonia, weak suck, and head lag. Additionally, the patient presented with a bell‐shaped thorax and knee flexion contractures. Hand deformities consisted of clenched hands with bilateral clinodactyly, camptodactyly, brachydactyly, short middle fingers, ulnar deviation of fingers and wrists, adducted thumbs, and abnormal digital flexion creases. Feet deformities included vertical talus, camptodactyly, brachydactyly, and short toes with broad halluces. Facial dysmorphism was also observed, featuring retrognathia, high arched palate, and low‐set and posteriorly rotated ears. In the first year of life, a mild delay in motor development was observed, with delayed age at sitting and walking. However, cognitive performances were within normal ranges. Follow‐up examination at the age of 4 years (Figure 1a) confirmed the presence of hypotonia, significant distal contractures of the hands and feet, flexion contractures of the knees, and obstructive sleep apnea. His development was appropriate for age.

### 3.2. Molecular Findings

ES led to the identification of variants in *TNNT3* in both subjects (Figure 1a). In Patient #1, the missense variant c.188G>A, p.(Arg63His), was detected. This change was confirmed to be absent in the parents and to have occurred de novo in the proband (Figure 1a, Figure S3). The p.(Arg63His) substitution is absent in gnomAD (V4.1.0) and affects a conserved residue (GERP score = 3.71) of the protein. Additionally, it is predicted pathogenic by several in silico tools (Table S1), being classified as pathogenic according to the American College of Medical Genetics and Genomics (ACMG) guidelines [26]. In Subject #2, two variants in compound heterozygous state were detected: the maternal c.480+5G>A and the paternal c.39C>G, p.(Tyr13∗) (Figure 1a, Figure S4). In gnomAD (V4.1.0), the c.480+5G>A variant is absent, whereas the p.(Tyr13∗) is present in heterozygous state in one subject (allele frequency = 0.00000398). Both variants affect conserved residues (GERP scores of 4.26 and 3.55, respectively). The c.480+5G>A variant affects a splicing donor site, and it is likely to alter splicing processes according to in silico predictions (Figure 1b, Table S1). The truncating variant p.(Tyr13∗) is predicted instead to lead to a nonsense‐mediated mRNA decay (NMD) or the formation of a truncated transcript. The c.480+5G>A is classified as a variant of unknown significance according to the ACMG guidelines, whereas the p.(Tyr13∗) is likely pathogenic. Overall, these two variants are predicted to cause a loss of TNNT3 protein function. The variants identified in our patients have been submitted to the Leiden Open Variation Database (LOVD, https://databases.lovd.nl) with the following accession numbers: #0000984937, #0000984938, and #0000984939.

### 3.3. p.(Arg63His) Destabilizes Troponin T3 Structure In Silico

The residue affected by the p.(Arg63His) substitution identified in Subject #1, Arg63, has been previously reported to be mutated in recurrent association with DA [8, 20]. The TNNT3 protein consists of 258 residues, spatially arranged into a single domain comprising three distinct alpha helices which are interspersed by unstructured regions (Figure 1c and Figure S5). Based on the AF2 local confidence metrics (pLDDT), the AF2‐TNNT3 model (Figure 1c) is of high quality (pLDDT > 90) for most of the residues forming alpha helices, with the exception of the segment located at the protein N‐terminus; disordered regions are characterized by lower pLDDT scores. The secondary structure assignment for D‐I‐TASSER‐TNNT3 corroborated the segment boundaries determination performed by AF2 (Figure 1c). The superimposition of the two predictions results in a root mean square deviation (RMSD) of 13.52 Å (Figure S5), with minor discrepancies detectable in helices’ spatial arrangement. These differences may be easily surpassed by the protein intrinsic dynamics. The mutated residue R63H/C/S (Figure 1c) is located at the N‐terminal region of the longer alpha helix, and it is flanked by positively charged residues which are grouped into a polar patch spanning from Q60 to K66 (Figure 1c). We calculated the *ΔΔ* *G* values for three mutants, R63H, R63S, and R63C, obtaining −0.61, −0.75, and −0.40 Kcal/mol, respectively. Hence, all three substitutions were predicted to perturb protein structure, confirming the functional relevance of the Arg63 residue.

To further characterize the impact of missense changes in Arg63, we constructed plasmids expressing p.R63H (c.188G>A) and p.R63C (c.187C>T) substitutions of TNNT3 fused to eGFP, as these variants are representative of the mutational hotspot in Arg63 in the TNNT3‐related disorder [2, 5, 6, 27] and transfected into HEK293 cells (Figure 1d and Figure S6). By western blot using an antibody directed against GFP, we observed an increased expression of two isoforms at 57.7 and 49.9 kDa (size of TNNT3 + eGFP) in the TNNT3 p.R63H (c.188G>A) and p.R63C (c.187C>T) variants compared to wt TNNT3‐GFP.

### 3.4. p.(Tyr13∗) Affects the Expression of the Full‐Length Troponin T3 Isoform

In Subject #2, the p.(Tyr13∗) is predicted to lead to no full‐length protein. To confirm this, we constructed plasmids expressing wt and p.Y13∗ (c.39C>G) variants of TNNT3 fused to eGFP (Figure 2a) and transfected into HEK293 cells. We performed a western blot analysis of TNNT3 p.Y13∗ fused to GFP expression using an antibody directed against GFP. While in the wt condition we observed a protein at 57.7 kDa (size of TNNT3 + eGFP, Figure S6), in the TNNT3 p.Y13∗ variant, we observed the absence of the 57.7 kDa protein (Figure 2a). This means that the full‐length TNNT3 isoform is not expressed with the p.Y13∗ which induces a stop codon. However, we observed a shorter isoform at 49.9 kDa (Figure 2b). Looking at the sequence, we identified a potential second start codon which could explain this shorter isoform (Figure 2c).

Figure 2Transfection assay with the TNNT3 p.(Tyr13∗) variant. The p.(Tyr13∗) variant results in the loss of the full‐length TNNT3 protein. (a) Both full‐length TNNT3 wild type and mutated c.39C>G versions were fused to GFP to monitor protein expression. (b) After transfection in HEK293T cells, GFP detection revealed two bands at 57.7 and 49.9 kDa for the wt constructs and only a truncated form of TNNT3‐GFP at 49.9 kDa for the p.Y13∗ variant. (c) Quantification of the two main bands of TNNT3‐GFP showing a clearance of the full‐length isoform and an increase in the shorter isoform. *T*‐test  ^∗∗^ *p* < 0.01. (d) While the c.39C>G variant leads to a stop codon, sequence analysis of TNNT3 revealed a potential second start codon in c.199 that could explain this shorter 49.9 kDa version.

**Figure:**
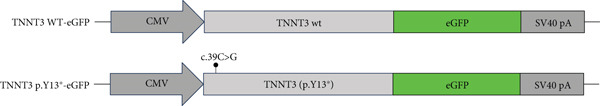
(a)

**Figure:**
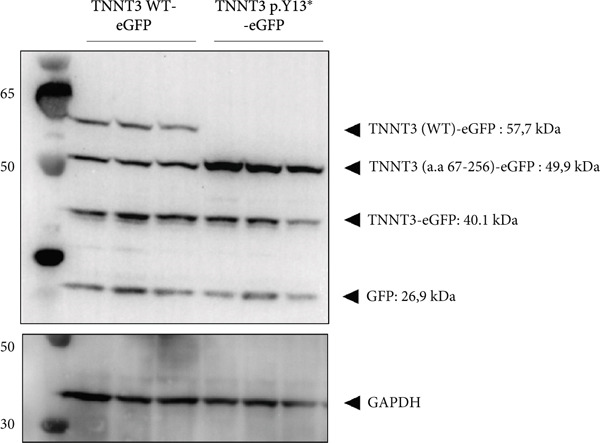
(b)

**Figure:**
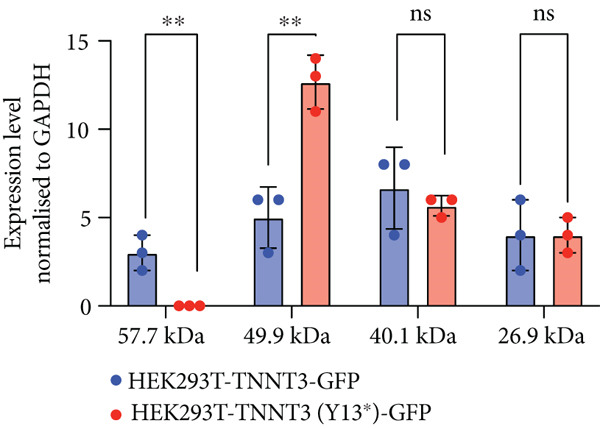
(c)

**Figure:**
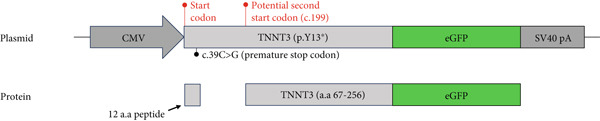
(d)

### 3.5. c.480+5G>A Leads to No Troponin T3 Protein Expression

The c.480+5G>A identified in Subject #2 affects a conserved donor site in Intron 12 of the *TNNT3* gene, and it is likely to lead to a splicing defect. To assess the consequences of this variant, we thus constructed plasmids expressing a minigene encompassing Exon 1–16 with the addition of Introns 10, 11, 12, and 13 with or without the c.480+5G>A intronic mutation between Exons 12 and 13 (Figure 3a) fused to GFP, and we transfected in HEK293T cells. By RT‐PCR, we observed a splicing defect in the mutated variant with the quasi absence of the spliced isoform (Figure 3b,c and Figure S7). The retention of Intron 12 as a result of this unspliced isoform leads to a premature stop codon. As a control, no splicing defect was observed in the near introns (Figure S8). To confirm this, we performed a western blot analysis comparing wt minigene and c.480+5G>A (intron) minigene fused to GFP expression using an antibody directed against GFP. While in the wt condition we observed the two isoforms at 57.7 and 49.9 kDa (size of TNNT3 + eGFP; Figure S6), in the TNNT3 c.480+5G>A (intron) variant, we observed the absence of both isoforms (Figure 3d). This means that TNNT3 protein is not expressed with the c.480+5G>A (intron) mutation due to the premature stop codon. These findings support the absence of a stable truncated protein resulting from the c.480+5G>A variant, likely due to nonsense‐mediated decay or protein instability, even under overexpression conditions. Previous studies, including [20], have reported different cellular localization of TNNT3. To further characterize the functional impact of the variants, we examined both the subcellular localization and expression levels of the fusion proteins by fluorescence microscopy, providing a complementary perspective to the western blot analysis. We analyzed GFP expression after transfection in human immortalized muscle cells of all the different TNNT3 constructs (p.[Arg63His] in Subject #1 and biallelic *TNNT3* variants p.[Tyr13∗] and c.480+5G>A in Subject #2) (Figure 4a). GFP expression from GFP‐fused constructs showed nonclassical nuclear localization where it formed sharp puncta in foci as previously described [20], and we observed GFP expression for all constructs except for the TNNT3 c.480+5G>A (intron) variant (Figure 4b).

Figure 3Minigene assay. The c.480+5G>A variant results in a splicing defect in Introns 12–13. (a) A minigene construct containing gDNA of TNNT3 between Exon 10 and Exon 14 (with intronic sequences) and cDNA of TNNT3 from Exons 1 to 10 and Exons 14 to 16 was designed. The c.480+5G>A variant was inserted in Intron 12. (b) After transfection in HEK293T cells, the splicing around Exons 12 and 13 of wild‐type TNNT3 minigene sequence was compared to the splicing in the mutated version using RT‐PCR. The variant led to change in the ratio of spliced versus unspliced form around Intron 12. (c) Quantification of band intensity revealed an almost complete abolition of the spliced form of Exons 12–13. (d) After transfection in HEK293T cells, GFP detection revealed two main isoforms for the wt minigene constructs and its complete absence for the c.480+5G>A variant minigene.

**Figure:**
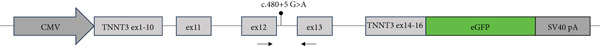
(a)

**Figure:**
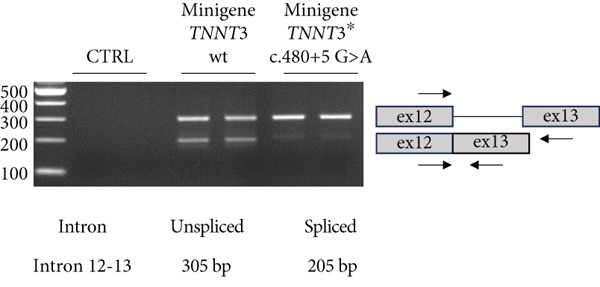
(b)

**Figure:**
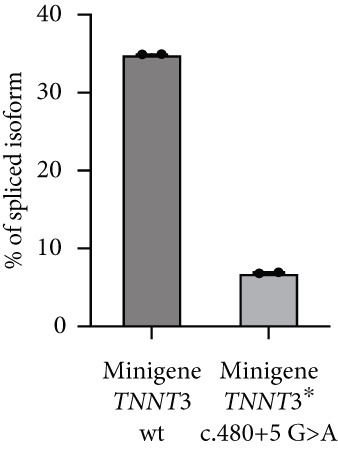
(c)

**Figure:**
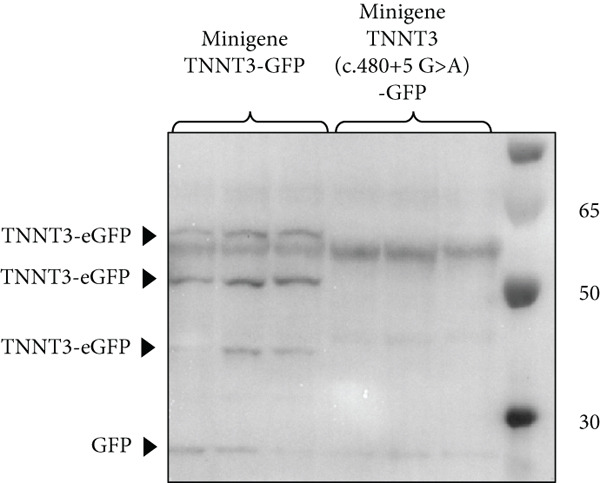
(d)

Figure 4(a, b) TNNT3‐GFP constructs expression in human muscle cells. Transfection of the different constructs harboring each variant and fused to eGFP in human immortalized muscle cells. For all constructs except the c.480+5G>A construct, protein expression is confirmed, with a nonclassical nuclear GFP expression in sharp puncta as previously described [21] (see inset box ×4). Objective 63×.

**Figure:**
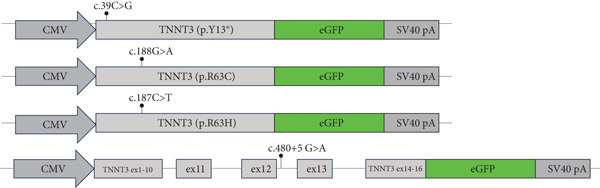
(a)

**Figure:**
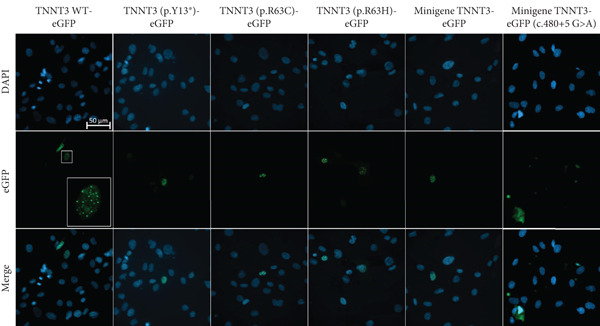
(b)

## 4. Discussion

In this study, we identified deleterious variants in *TNNT3* segregating in dominant (de novo) or recessive patterns in two unrelated patients from two unrelated families, presenting with myopathic manifestations at the different phenotypic ends of the *TNNT3* spectrum. Subject #1 presented with a dominant DA, whereas Subject #2 displayed a recessive congenital myopathy. The recessive *TNNT3* variants were found to significantly impact protein levels, supporting a distinctive loss of function mechanism underlying the emerging *TNNT3*‐related congenital myopathy. While dominant deleterious *TNNT3* variants are a well‐known cause of DA, the association of biallelic deleterious changes and congenital myopathy is very recent, with only two cases reported so far [12, 13]. The first patient was a newborn harboring the homozygous c.681+1G>A variant and presenting with a nemaline myopathy and DA [12]. The second case was a 4‐year‐old patient harboring the homozygous c.481‐1G>A variant and showing severe myopathic involvement, featuring myopathic facies, hypotonia, and respiratory insufficiency [13]. Of note, this patient showed an overall mild impairment of motor development compared to other previously reported cases [13], possibly involving the persistence of some function thanks to the production of a shorter isoform. Overlapping clinical features were observed in Subject #2 in our study, who harbored biallelic truncating variants in *TNNT3* (Table 1). In particular, the presence of DA, facial and limb weakness, hypotonia with decreased reflexes, and respiratory involvement appear to be distinctive of *TNNT3*‐related congenital myopathy in comparison to DA2B2. Additional features observed in this condition that are instead rare in DA2B2 include myopathic facies and scoliosis. Although only a very limited number of patients harboring biallelic deleterious variants in *TNNT3* have been reported so far, the emerging core clinical phenotype suggests that this congenital myopathy should be considered a discrete entity, with only partial overlap with DA2B2. The report of additional cases will play a crucial role in expanding the phenotypic spectrum of *TNNT3*‐related congenital myopathy and better delineate the phenotypic continuum with DA2B2.

**Table 1:** Genetic and clinical features of patients with recessive *TNNT3*‐related myopathy.

	**This report (Subject #2)**	**Sandaradura et al., [** **12** **]**	**Calame et al., [** **13** **]**	**Recessive TNNT3-related congenital myopathy**	**Dominant DA2B2**
*TNNT3* variant(s) (NM_006757.4)	[c.480+5G>A; c.39C>G, p.(Tyr13∗)]	c.681+1G>A	c.481‐1G>A	Biallelic deleterious variants	Heterozygous deleterious variants
Distal arthrogryposis	Yes	Yes	Yes	Yes (3/3)	Yes
Hypotonia	Yes	Yes	No	Yes (2/3)	No
Myopathic facies	No	Yes	Yes	Yes (2/3)	No
Triangular facies	Yes	No	No	Yes (1/3)	Yes
Facial weakness	Yes	Yes	Yes	Yes (3/3)	No
Limb weakness	Yes	Yes	Yes	Yes (3/3)	No
Bulbar involvement	Yes	Yes	Yes	Yes (3/3)	No
Respiratory involvement	Yes	Yes	Yes	Yes (3/3)	No
Absent reflexes	Yes	Yes	Yes	Yes (3/3)	No
Scoliosis	No	Yes	Yes	Yes (2/3)	No

The genetic investigation of the subjects enrolled in our study led to the identification of distinctive *TNNT3* genotypes. Subject #1 was found to harbor a recurring de novo missense change affecting Arg63. The de novo status was confirmed with Sanger sequencing (Figure S3) and sample relatedness analysis confirmed the biological parentship (Figure S9). Arg63 is located in a highly conserved region (corresponding to tropomyosin Binding Site 1), and it is often mutated in autosomal dominant DA2B2 [2, 5, 6, 28]. The same p.(Arg63His) substitution identified in our patient has been previously identified in a large Indian family with 18 affected members showing features of DA2B2 [10]. Another variant in the same residue, p.(Arg63Cys), was instead reported in association with features of Sheldon–Hall syndrome (SHS), also known as DA2B, in multiple members of a Chinese family with vertical talus [6]. Compared to DA2B2, SHS is characterized by a more prominent dysmorphism and more variable presence of vertical talus [2, 4, 5]. Although the clinical overlap between these two conditions may complicate differential diagnosis, these observations suggest that different variants involving Arg63 may result in subtle divergences in phenotypic expression within the DA2B‐DA2BS spectrum.

The p.(Arg63His) variant showed a significant destabilizing impact on the structure of the TNNT3 protein in silico, comparable to the consequences of other previously reported deleterious variants affecting this highly conserved residue, including p.(Arg63Cys) [2, 5, 6, 20]. By western blot using expression vectors, we observed an increased stability with both p.R63H (c.188G>A) and p.R63C (c.187C>T) substitutions. Substitutions affecting Arg63 may be recurrent in either DA2B2‐ or *TNNT3*‐related congenital myopathy. Thus, further studies may be helpful to identify potential additional molecular mechanisms of novel human substitutions involving Arg63, beyond the biochemical properties of the p.(Arg63His) [7], to delineate the functional spectrum of variants affecting this critical residue. Subject #2 harbored instead recessive variants in *TNNT3*, featuring a truncating and a splicing change occurring in trans. As such, this is the first patient reported to harbor compound heterozygous variants in *TNNT3*, and the p.(Tyr13∗) is the first stop gain variant identified in *TNNT3*‐related congenital myopathy. Furthermore, the c.480+5G>A is another splicing variant to be associated with this condition, in which splicing defects are present in most cases reported so far (Table 1).

Two distinct variants were reported in previous patients with recessive TNNT3‐related myopathy, the c.481‐1G>A and c.681+1G>A variants [12, 13]. Both these variants are predicted to be deleterious due to the disruption of an acceptor and donor splice site, respectively [12, 13]. As a result, these variants are likely to result in the formation of a truncated transcript or result in NMD, with evidence of a significant impact on protein levels in patient muscle tissue [13]. Our studies showed that the p.(Tyr13∗) and c.480+5G>A variants both impact full‐length troponin T3 function. Both these variants significantly affect full‐length protein levels. The paternal p.(Tyr13∗) variant leads to decreased full‐length protein expression, while shorter isoforms are still present. This truncated shorter isoform of the troponin T3 protein likely results from the use of a second distal start codon. The maternal c.480+5G>A variant causes instead a complete absence of the protein, as a consequence of a severe disruption of the splicing process and a premature stop codon. Although SpliceAI predictions may lead to suggest that there might be a cryptic donor gain and the spliced product in the variant minigene construct may not be the wt sequence, this tool is based on in silico predictions and may not reflect the actual functional consequences of the splicing variant investigated here. In fact, Sanger sequencing data confirmed the identity of the splicing products for the wt and the variant (supporting information). Comprehensively, these findings refine and expand the molecular spectrum of *TNNT3*‐related myopathic manifestations, suggesting that variants leading to the loss of full‐length TNNT3 protein while maintenance of shorter truncated transcripts underlie recessive cases.

Our study has some limitations. Subject #1 is an aborted fetus, which limited the extent of the clinical information available. However, the identification of a de novo missense change affecting a recurrently mutated residue (Arg63) in DA2B strongly supports this diagnosis. The p.(Tyr13∗) variant was inherited by the father of Subject #2, who had a history of bilateral clubfeet. The TNNT3 haploinsufficiency resulting from this change may have contributed to the clubfoot anomaly, but, unfortunately, it was not possible to explore the segregation of this variant in paternal relatives. However, no member other than the proband’s father was reported to have musculoskeletal defects, making a carrier status unlikely. The father of Subject #2 did not receive specific genetic testing or other clinical investigations to rule out other potential causes of clubfeet. We could not confirm or exclude the presence of abnormal muscle histology (i.e., nemaline myopathy or fatty infiltration) in the reported subjects. Although our findings on subcellular localization are consistent with those reported for the endogenous protein in previous work [20], we cannot fully exclude the possibility that the GFP fusion system affects the localization or behavior of the protein variants. As such, our study does not allow us to assess potential (mis)localization in the endogenous context, particularly in patient‐derived cells. The interpretation of the p.(Tyr13∗) is also limited by the use of cDNA which does not reflect the genomic context of TNNT3 and the possibility of NMD. As such, the complexity and wide range of *TNNT3* variants make it difficult to understand the respective contribution and function of each variant and therefore the physiological consequences in each case. Another limitation concerns the cellular models used. While HEK293T cells provided a practical system to assess protein stability and detectability, they do not recapitulate the contractile environment of myocytes. To increase physiological relevance, we also examined expression and localization in human myoblasts. However, functional aspects such as myofilament incorporation or downstream effects were not explored and will require dedicated future studies.

In this study, we investigated two patients presenting with either dominant or recessive forms of *TNNT3*‐related myopathic involvement. Through ES, we identified a de novo variant in a highly mutated residue in DA2B2 in Subject #1 and biallelic truncating and splice variants in Subject #2. We showed that the p.(Arg63His) substitution affects troponin T3 structure stability in silico and in vitro, whereas the p.(Tyr13∗) and c.480+5G>A variants significantly impacted protein expression. Our findings expand the *TNNT3* genotype and phenotype spectrum, suggesting that variants resulting in a severe loss of protein function underlie the recessive *TNNT3*‐related congenital myopathy. Future studies will be crucial to further delineate the pathophysiological mechanisms involved in the emerging myopathy.

## Ethics Statement

This study adheres to the principles in the Declaration of Helsinki. The study was reviewed by IRCCS Istituto Giannina Gaslini Review Board (IRB) (Comitato Etico della Regione Liguria, Protocol 163/2018). Written informed consent was obtained from all participants including consent for publication of photographs as required by the IRB. Consent forms are archived and available upon request.

## Conflicts of Interest

J.P. is an employee of GeneDx, LLC. The remaining authors declare no conflicts of interest.

## Author Contributions

Conceptualization: M.S.; data curation: A.B., M.S., C.T., N.A.; formal analysis: M.S., C.T., N.A., K.M., J.O., B.C., L.M.; data collection: F.M., M.O., M.S.S.N., D.P., M.I., A.R., O.B., S.Q., J.P., V.C., F.Z.; methodology: M.S., C.T.; writing—original draft: M.S., C.T., N.A.; writing—review and editing: M.S., C.T.; supervision: M.S., C.T.

## Funding

This work was financed by INSERM, Sorbonne University, the Association Institut de Myologie, and the Fondation Recherche Médicale. This research was also supported by PNRR‐MUR‐M4C2 PE0000006 Research Program “MNESYS”—a multiscale integrated approach to the study of the nervous system in health and disease.

## Supporting information


**Supporting Information** Additional supporting information can be found online in the Supporting Information section. Supporting Methods: Detailed description of the experimental methodologies used in the study, including participant enrollment from various research centers, exome sequencing protocols, Sanger sequencing validation, and in silico analyses of candidate variants. Specific computational tools and parameters for genomic and splicing studies are also provided. Table S1: Summary of in silico predictions for TNNT3 variants, including computational annotations and predicted impacts. Table S2: Primer sequences used for various PCR and sequencing experiments, including cloning and splicing studies. Figure S1: (A) GTEX representation (https://www.gtexportal.org/home/gene/TNNT3) of both TNNT3 transcripts NM_006757.4 and TNNT3‐207 (NM_001042781.3) used in the study. The transcript NM_006757.4 is the canonical transcript used for the diagnostic. Other TNNT3 transcripts are expressed in muscle, among which the TNNT3‐207 (NM_001042781.3) is the most expressed one in muscle cells. There is a strong homology between both isoforms at the RNA (B) and protein (C) levels, and this does not modify the impact of the genetic mutations described in the study. Figure S2: Clinical photographs of the parents of Subject #2, highlighting musculoskeletal phenotypes. Figure S3: Electropherogram from Sanger sequencing showing the de novo occurrence of the TNNT3 variant p.(Arg63His) in Subject #1. Figure S4: IGV snapshot demonstrating the compound heterozygous inheritance of variants in Subject #2. Figure S5: Structural superimposition of TNNT3 predictions using AF2 and D‐I‐TASSER, highlighting structural differences and confidence levels. Figure S6: Scheme of the TNNT3‐GFP construct. Figure S7: Clustal alignment of unspliced and spliced band from the minigene assay. Figure S8: Gel electrophoresis results from minigene assays, showing a splicing defect in Intron 12 but no defects in adjacent introns. Supporting References: A curated list of all references cited in the supporting information, including key genomic and bioinformatics methodologies and related studies. Figure S9: This heatmap was generated using Somalier, a tool commonly employed in genomic analysis to assess sample relatedness and detect contamination by analyzing kinship and ancestry from alignment files. The figure displays an allele‐sharing matrix in which the color of each cell represents the relationship between two samples. Dark red along the diagonal indicates identical samples or self‐comparisons, while off‐diagonal dark red suggests possible sample swaps or significant cross‐sample contamination. Orange tones reflect first‐degree relationships such as parent–child or full siblings. Blue tones denote unrelated or distantly related individuals. The heatmap was visualized using the MultiQC platform.

## Data Availability

All data described in this study are provided within the article and supporting information. Raw sequencing data and deidentified clinical data is available from the corresponding authors upon request.
