# Supplementary material for: The Emerging TNNT3 Spectrum: From Distal Arthrogryposis to Congenital Myopathy
Source: Hum Mutat. 2025 Dec 28;2025:1785045. doi: 10.1155/humu/1785045 (PMC12745331; doi:10.1155/humu/1785045)
Supplement: Supplementary file 1 — Supporting Information Additional supporting information can be found online in the Supporting Information section. Supporting Methods: Detailed description of the experimental methodologies used in the study, including participant enrollment from various research centers, exome sequencing protocols, Sanger sequencing validation, and in silico analyses of candidate variants. Specific computational tools and parameters for genomic and splicing studies are also provided. Table S1: Summary of in silico predictions for TNNT3 variants, including computational annotations and predicted impacts. Table S2: Primer sequences used for various PCR and sequencing experiments, including cloning and splicing studies. Figure S1: (A) GTEX representation (https://www.gtexportal.org/home/gene/TNNT3) of both TNNT3 transcripts NM_006757.4 and TNNT3‐207 (NM_001042781.3) used in the study. The transcript NM_006757.4 is the canonical transcript used for the diagnostic. Other TNNT3 transcripts are expressed in muscle, among which the TNNT3‐207 (NM_001042781.3) is the most expressed one in muscle cells. There is a strong homology between both isoforms at the RNA (B) and protein (C) levels, and this does not modify the impact of the genetic mutations described in the study. Figure S2: Clinical photographs of the parents of Subject #2, highlighting musculoskeletal phenotypes. Figure S3: Electropherogram from Sanger sequencing showing the de novo occurrence of the TNNT3 variant p.(Arg63His) in Subject #1. Figure S4: IGV snapshot demonstrating the compound heterozygous inheritance of variants in Subject #2. Figure S5: Structural superimposition of TNNT3 predictions using AF2 and D‐I‐TASSER, highlighting structural differences and confidence levels. Figure S6: Scheme of the TNNT3‐GFP construct. Figure S7: Clustal alignment of unspliced and spliced band from the minigene assay. Figure S8: Gel electrophoresis results from minigene assays, showing a splicing defect in Intron 12 but no defects in adj [file HUMU-2025-1785045-s001.zip › Supplementary Material.docx]

**Supplementary Material**

**1. Supplementary Methods**

**2. Supplementary Table**

**3. Supplementary Figure**

**4. Supplementary References**

**1. Supplementary Methods**

***1.1 Participants enrolment***

The patients investigated in this study were enrolled at several different international research centers and hospitals: IRCCS Istituto Giannina Gaslini, Genova, Italy; Division of Genetics and Genomics, Department of Medicine, Boston Children's Hospital/Harvard Medical School, United States.

***1.2 Exome sequencing analysis***

After standard DNA extraction, trio-exome sequencing (ES) was performed as previously described (1-5). Statistics with FastQC (http://www.bioinformatics.bbsrc.ac.uk/projects/fastqc) was used to assess the quality of the sequence reads. BWA with default parameters was used for reads alignment to the reference human genome (GRCh38 - hg38, UCSC genome assembly). Recalibration of the quality score and for indel realignment and variant calling was performed through the HaplotypeCaller algorithm within the GATK package (6, 7). Variants were then annotated with ANNOVAR (8) and filtered out for minor allele frequency (MAF) ≤ 0.01 in genomic databases (GnomAD, <https://gnomad.broadinstitute.org>). Afterwards, *in silico* tools were employed to predict the impact of candidate variants on protein structure and function, including: Combined Annotation Dependent Depletion (CADD, <https://cadd.gs.washington.edu>), Mutation Taster (<http://www.mutationtaster.org>), Mutation Assessor (<http://mutationassessor.org/r3/>), Polyphen-2 (<http://genetics.bwh.harvard.edu/pph2/>), and Splice AI (<https://spliceailookup.broadinstitute.org>). Sanger sequencing was performed according to standard procedures^5^ to confirm the most plausible candidate variants and for parental segregation analysis. For subject #2, ES protocols have been previously described (8) and variants reported by GeneDx were confirmed by an orthogonal method, as appropriate.

***1.3 Sanger sequencing***

Candidate variants in *TNNT3* were validated by Sanger sequencing using High-Fidelity Platinum Master Mix (Thermo Fisher Scientific) for PCR amplification and the BigDye Terminator v1.1 kit (Thermo Fisher Scientific) for sequencing.

***1.4 Sanger sequencing of minigene products***

Sanger sequencing of the minigene products was performed to confirm the exact sequences for the spliced and unspliced products (see Figure S7 below).

***1.4 Sample relatedness analysis***

To confirm familial relationships and assess potential sample contamination, we employed Somalier (Pedersen et al., 2020), a computational tool widely used in genomic studies for relatedness inference. Somalier analyzes kinship and ancestry using genotype data derived from alignment (BAM/CRAM) files. The analysis generated a relatedness heatmap, which demonstrated high kinship coefficients between the probands and their respective parents, consistent with first-degree biological relationships. No evidence of sample contamination or sample swaps was detected.

**2. Supplementary Tables**

**Table S1. *In silico* analysis of *TNNT3* variants.**

**Table S2. Primers used in the study**

|  | Primers | |
| --- | --- | --- |
| PCR | Forward | Reverse |
| TNNT3 cDNA cloning | GATCTCGAGCTCAAGCTTCGATGTCTGACGAGGAAGTTG | TGATCAACCGGTCCCTTCCAGCGCCCGCCGACTTT |
| TNNT3 Minigene fragment 1 cloning | GATCTCGAGCTCAAGCTTCGATGTCTGACGAGGAAGTTG | CCCCACTCACGATTCTCTCTTTGAGAGCG |
| TNNT3 Minigene fragment 2 cloning | AGAGAGAATCGTGAGTGGGGCAGTTCAG | GGCCTTGTCCCTGGGGCAGAGGAAGTGG |
| TNNT3 Minigene fragment 3 cloning | TCTGCCCCAGGGACAAGGCCAAGGAGCTCTGG | CTTGCTCACCATGGTGGCGAACTTCCAGCGCCCGCCGA |
| Intron 12-13 splicing study | GAGGAGGAGGATGCCAAGAG | TCAGTTTGTCTTCACCAAGGTG |
| Intron 11-12 splicing study | TTCGTGCAGAGAAGGAGAGG | CTCCCATGGAAGACAGAGCT |
| Intron 13-14 splicing study | AGCCCGGGAAATGAAGAAGA | AGCTTCTCCCCAAACTCGAA |
| Intron 10-11 splicing study | CCTCATCGACAGCCACTTTG | CCTCTCCTTCTCTGCACGAA |

**3. Supplementary Figures**

A.

B.

C.

**Figure S1:** **TNNT3 isoforms.** A. GTEX representation (https://www.gtexportal.org/home/gene/TNNT3) of both TNNT3 transcripts NM_006757.4 and TNNT3-207 (NM_001042781.3) used in the study. The transcript NM_006757.4 is the canonical transcript used for the diagnostic. Other TNNT3 transcripts are expressed in muscle, among which the TNNT3-207 (NM_001042781.3) is the most expressed one in muscle cells. There is a strong homology between both isoforms at the RNA (B.) and protein (C.) levels and this does not modify the impact of the genetic mutations described in the study.

**Figure S2. Clinical photographs of the parents of subject #2.** The mother (**A**) of #2 does not show significant musculoskeletal abnormalities, whereas a bilateral clubfoot can be observed in the father (**B**).

**Figure S3. Sanger sequencing.** DNA sequence electropherogram showing that the p.(Arg63His) variant in *TNNT3* occurs *de novo* in subject #1.

**Figure S4. IGV snapshot of subject #2.** This individual inherited the variants in compound heterozygous pattern from his unaffected parents, of whom the mother harbors the c.480+5G>A and the father harbors the c.39C>G, p.(Tyr13*).

**
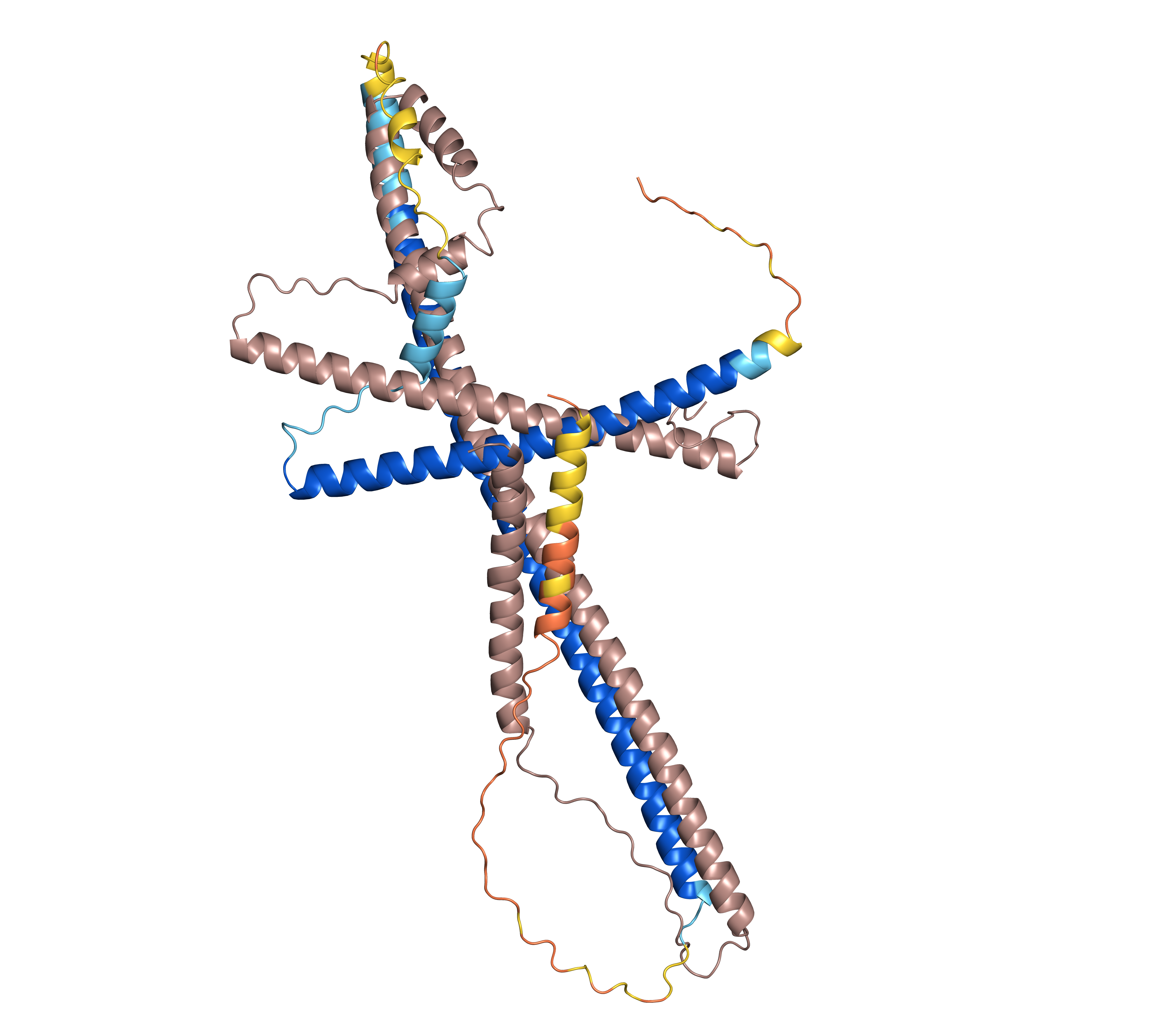
**

**Figure S5. Superimposition of AF2-TNNT3 and D-I-TASSER-TNNT3 predictions.** The structural models are shown in cartoons; while AF2-TNNT3 is coloured by pLDDT, D-I-TASSER-TNNT3 is portrayed in dark red.

**Figure S6: Scheme of the TNNT3-GFP construct** including the alternative start codons and the Tropomyosin-, TnC and TnI binding sites (based on ref: Wei B, Jin JP. TNNT1, TNNT2, and TNNT3: Isoform genes, regulation, and structure-function relationships. Gene. 2016 May 10;582(1):1-13. doi: 10.1016/j.gene.2016.01.006. Epub 2016 Jan 13. PMID: 26774798; PMCID: PMC5325693.)

**Figure S7.** Clustal alignment as a result of the Sanger sequencing on both wild-type and variant spliced bands.


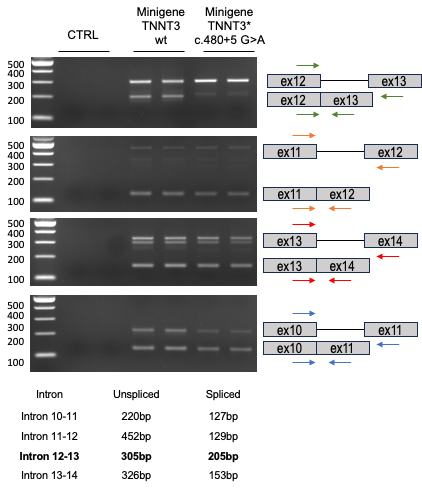


**Figure S8. Minigene assay.** While splicing defect was observed in intron 12 (green, top gel), no splicing defect were observed in nearby introns 10, 11 and 13 as revealed by gel electrophoresis of RT-PCR surrounding each intron.

**
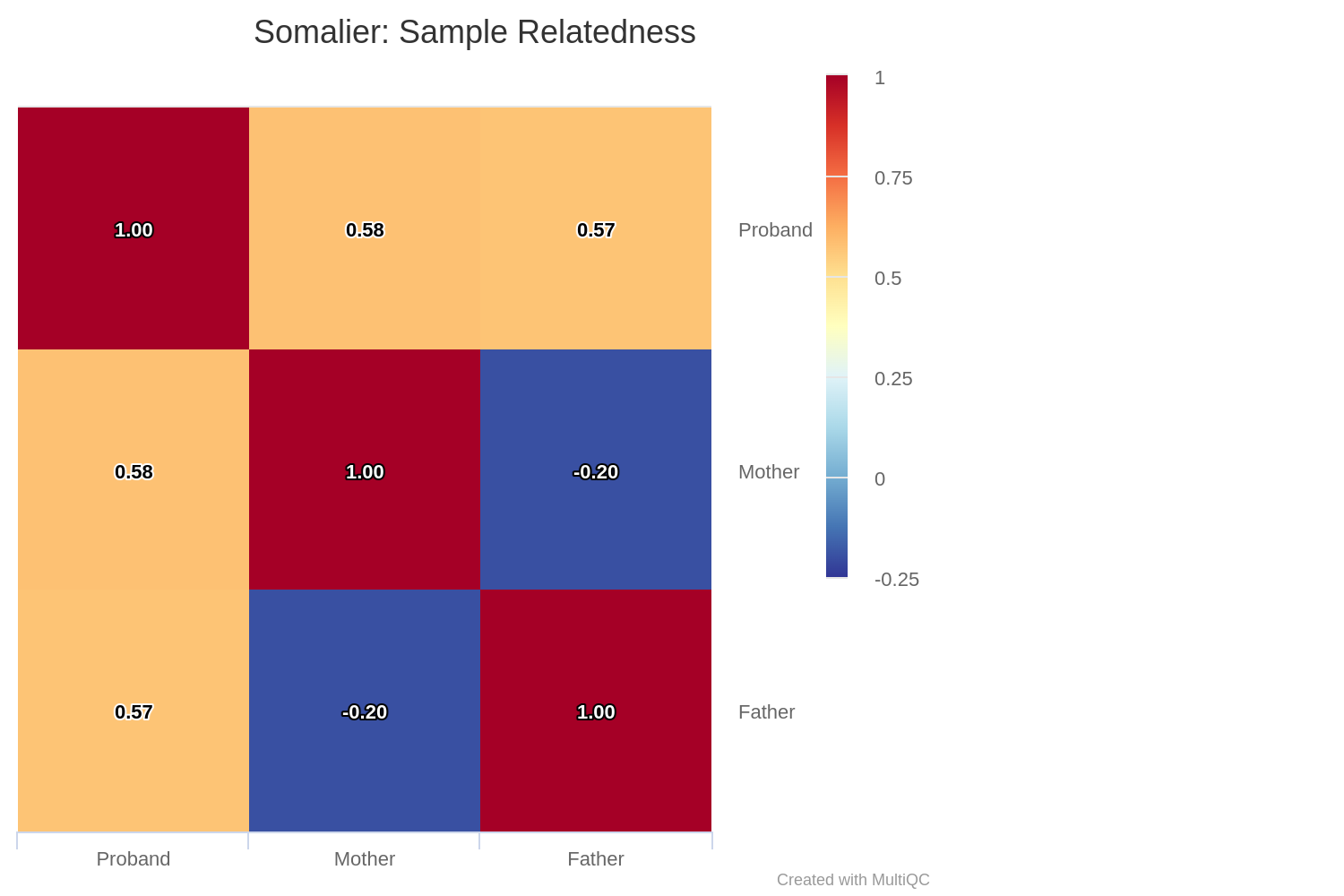
**

**Figure S9. Kinship and Contamination Heatmap.** This heatmap was generated using Somalier, a tool commonly employed in genomic analysis to assess sample relatedness and detect contamination by analyzing kinship and ancestry from alignment files. The figure displays an allele-sharing matrix in which the color of each cell represents the relationship between two samples. Dark red along the diagonal indicates identical samples or self-comparisons, while off-diagonal dark red suggests possible sample swaps or significant cross-sample contamination. Orange tones reflect first-degree relationships such as parent–child or full siblings. Blue tones denote unrelated or distantly related individuals. The heatmap was visualized using the MultiQC platform.

**4. Supplementary References**

1. Aspromonte MC, Bellini M, Gasparini A et al. Characterization of intellectual disability and autism comorbidity through gene panel sequencing. Hum Mutat 2019;40:1346-1363. doi: 10.1002/humu.23822.

2. Bowling KM, Thompson ML, Amaral MD, et al. Genomic diagnosis for children with intellectual disability and/or developmental delay. Genome Med. 2017;9:43. doi: 10.1186/s13073-017-0433-1.

3. Harripaul R, Noor A, Ayub M, Vincent JB. The Use of Next-Generation Sequencing for Research and Diagnostics for Intellectual Disability. Cold Spring Harb Perspect Med. 2017; 7:a026864. doi: 10.1101/cshperspect.a026864.

4. Murdock DR, Dai H, Burrage LC, et al. Transcriptome-directed analysis for Mendelian disease diagnosis overcomes limitations of conventional genomic testing. J Clin Invest. 2021; 131:e141500. doi: 10.1172/JCI141500.

5. Tarailo-Graovac M, Shyr C, Ross CJ, et al. Exome Sequencing and the Management of Neurometabolic Disorders. N Engl J Med. 2016;374:2246-55. doi: 10.1056/NEJMoa1515792.

6. De Pristo MA, Banks E, Poplin R, et al. A framework for variation discovery and genotyping using next-generation DNA sequencing data. Nat Genet. 2011;43:491-498. doi: 10.1038/ng.806.

7. McKenna A, Hanna M, Banks E, et al. The Genome Analysis Toolkit: a MapReduce framework for analyzing next-generation DNA sequencing data. Genome Res. 2010; 20:1297–303. doi:10.1101/gr.107524.110

8. Wang K, Li M, Hakonarson H. ANNOVAR: functional annotation of genetic variants from high- throughput sequencing data. Nucleic Acids Res. 2010; 38:e164. doi:10.1093/nar/gkq603

9. Guillen Sacoto MJ, Tchasovnikarova IA, Torti E, Forster C, Andrew EH, Anselm I, Baranano KW, Briere LC, Cohen JS, Craigen WJ, Cytrynbaum C, Ekhilevitch N, Elrick MJ, Fatemi A, Fraser JL, Gallagher RC, Guerin A, Haynes D, High FA, Inglese CN, Kiss C, Koenig MK, Krier J, Lindstrom K, Marble M, Meddaugh H, Moran ES, Morel CF, Mu W, Muller EA 2nd, Nance J, Natowicz MR, Numis AL, Ostrem B, Pappas J, Stafstrom CE, Streff H, Sweetser DA, Szybowska M; Undiagnosed Diseases Network; Walker MA, Wang W, Weiss K, Weksberg R, Wheeler PG, Yoon G, Kingston RE, Juusola J. De Novo Variants in the ATPase Module of MORC2 Cause a Neurodevelopmental Disorder with Growth Retardation and Variable Craniofacial Dysmorphism. Am J Hum Genet. 2020 Aug 6;107(2):352-363. doi: 10.1016/j.ajhg.2020.06.013. Epub 2020 Jul 20. PMID: 32693025; PMCID: PMC7413887.

Wei B, Jin JP. TNNT1, TNNT2, and TNNT3: Isoform genes, regulation, and structure-function relationships. Gene. 2016 May 10;582(1):1-13. doi: 10.1016/j.gene.2016.01.006. Epub 2016 Jan 13. PMID: 26774798; PMCID: PMC5325693

10. Pedersen BS, Bhetariya PJ, Brown J, Kravitz SN, Marth G, Jensen RL, Bronner MP, Underhill HR, Quinlan AR. Somalier: rapid relatedness estimation for cancer and germline studies using efficient genome sketches. Genome Med. 2020 Jul 14;12(1):62. doi: 10.1186/s13073-020-00761-2. PMID: 32664994; PMCID: PMC7362544.
